# Effects of Multi-Generational Stress Exposure and Offspring Environment on the Expression and Persistence of Transgenerational Effects in *Arabidopsis thaliana*

**DOI:** 10.1371/journal.pone.0151566

**Published:** 2016-03-16

**Authors:** Maartje P. Groot, Rik Kooke, Nieke Knoben, Philippine Vergeer, Joost J. B. Keurentjes, N. Joop Ouborg, Koen J. F. Verhoeven

**Affiliations:** 1 Experimental Plant Ecology, Institute for Water and Wetland Research, Radboud University Nijmegen, Nijmegen, The Netherlands; 2 Department of Plant Physiology, Wageningen University, Wageningen, The Netherlands; 3 Plant Ecology and Nature Conservation Group, Wageningen, The Netherlands; 4 Department of Genetics, Wageningen University, Wageningen, The Netherlands; 5 Netherlands Institute of Ecology (NIOO-KNAW), Department of Terrestrial Ecology, Wageningen, The Netherlands; 6 Centre for BioSystems Genomics, Wageningen, The Netherlands; Blaustein Institutes for Desert Research, Ben-Gurion University of the Negev, ISRAEL

## Abstract

Plant phenotypes can be affected by environments experienced by their parents. Parental environmental effects are reported for the first offspring generation and some studies showed persisting environmental effects in second and further offspring generations. However, the expression of these transgenerational effects proved context-dependent and their reproducibility can be low. Here we study the context-dependency of transgenerational effects by evaluating parental and transgenerational effects under a range of parental induction and offspring evaluation conditions. We systematically evaluated two factors that can influence the expression of transgenerational effects: single- versus multiple-generation exposure and offspring environment. For this purpose, we exposed a single homozygous *Arabidopsis thaliana* Col-0 line to salt stress for up to three generations and evaluated offspring performance under control and salt conditions in a climate chamber and in a natural environment. Parental as well as transgenerational effects were observed in almost all traits and all environments and traced back as far as great-grandparental environments. The length of exposure exerted strong effects; multiple-generation exposure often reduced the expression of the parental effect compared to single-generation exposure. Furthermore, the expression of transgenerational effects strongly depended on offspring environment for rosette diameter and flowering time, with opposite effects observed in field and greenhouse evaluation environments. Our results provide important new insights into the occurrence of transgenerational effects and contribute to a better understanding of the context-dependency of these effects.

## Introduction

In plants phenotypic changes are determined not only by the environment, genotype and their interactions but also by the phenotype or the environment of the parents (i.e., parental effects) [1–9]. While ample evidence has been found for parental effects [4, 5, 7, 8], recent studies suggest that phenotypic responses to environmental conditions can also persist over multiple offspring generations (transgenerational effects, i.e. effects of environments experienced by grandparents or even earlier generations). Environment-induced transgenerational effects have been reported for both plant (e.g., *Arabidopsis thaliana* and *Mimulus guttatus*), and animal species (e.g., *Caenorhabditis elegans*, *Folsomia candida* and *Mus musculus*) and for different biotic and abiotic environmental treatments [10–19]. For example, in *A*. *thaliana* exposure to herbivores in one generation led to an increased resistance to herbivores in two subsequent generations [14]. Similar results were found for exposure to a pathogen [15]. When *A*. *thaliana* was exposed to multiple generations of heat treatment the offspring showed a higher reproductive output when grown in heat treatment compared to offspring that was grown in unheated environments [13]. Ancestral exposure of *A*. *thaliana* to salt stress resulted in improved growth under salt stress [16], while another study showed, for some genotypes, bigger leaves and rosette diameter when offspring from salt treated ancestors were grown under salt stress [17, 18].

Despite these empirical observations, several issues remain unsolved. Most important perhaps is that transgenerational effects are not always consistently observed and some effects could not be reproduced, raising doubts about their consistency [20, 21]. For example, Rasmann et al (2012) performed nine independent experiments to test for increased resistance to herbivory after ancestral exposure to herbivores. Only in seven out of nine experiments an increase in transgenerational resistance to herbivory was found [14]. Comparisons and generalisations between studies are complicated by the facts that the expression of transgenerational effects is sensitive to timing, duration, and severity of the environmental factor [16]. Besides, phenotypic responses may also vary between genotypes and traits [18, 22].

In addition, recent studies revealed evidence that expression of transgenerational effects can be affected by the number of consecutive generations of exposure to an environmental factor. For instance, in the nematode *C*. *elegans*, a stable transmission of odour receptiveness for more than 40 offspring generations was induced after multiple consecutive generations of exposure to an odour cue, whereas no such responses evolved after exposure of a single generation to stress [23]. Likewise, *A*. *thaliana* plants express a different phenotype after heat exposure of three consecutive generations as compared to plants that were exposed to heat for only one or two consecutive generations [17]. These findings suggest that transgenerational effects may be enhanced by the exposure of multiple consecutive generations to an environmental factor (i.e. dose effects) [17] and environmental shifts—when stable over multiple generations—may lead to transgenerational effects [24]. However, these dose effects are still little explored [18]. Furthermore, the expression of parental and transgenerational effects strongly depends on the offspring environment and whether this resembles the maternal environmental conditions [4, 13, 17].

These contrasting results hinder generalisations regarding the occurrence (and their ecological or evolutionary impacts) of parental effects, especially in field environments where multiple factors apply. To evaluate the context-dependency of the expression of transgenerational effects we chose to study salt stress responses in *A*. *thaliana*. Earlier studies indicated the existence of parental effects of salt stress for this species [16, 25], as well as transgenerational effects [17, 18, 26]. However, these effects appeared to be very context-dependent and were shown only under some experimental conditions and in some genotypes [16–18, 20].

Our study presents a systematic evaluation of two factors that may be involved in the context-dependency of parental and transgenerational effects. Specifically, the aim of our study was to evaluate these parental and transgenerational effects in *A*. *thaliana* after exposure of a variable number of ancestral generations to salt stress in different offspring environments. A single accession (homozygous, inbred line of *A*. *thaliana* Col-0) was used and a number of key traits were measured in multiple environments [27–29]. Our unique full factorial experimental setup allowed us to test for different effects of single versus multiple-generations of ancestral stress exposure, up to three generations. Furthermore we evaluated offspring phenotypes from the same experimental design in three different environments, including a field environment, to test for reproducibility and consistency among environments. The following questions were addressed: Are there parental and transgenerational environment-induced responses in *A*. *thaliana*? Is there a dose effect after exposure for multiple consecutive generations? And is the expression of transgenerational effects affected by offspring environment?

## Materials and Methods

### Parental treatments

**Fig 1:**
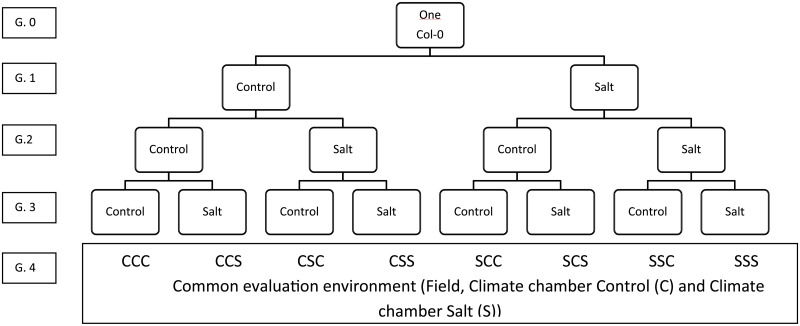
Origin of the experimental groups. A single *A*. *thaliana* plant (ecotype Col-0) served as a founder for the pedigree. Plants were grown for three generations either in a salt or in a control environment. Performance of offspring of the third generation (i.e., G.4) was tested in three distinct environments: a field environment, a climate chamber control and climate chamber salt environment.

Seeds of a single *A*. *thaliana* ecotype Col-0 were stratified for five days at 4°C on filter paper that was saturated with deionised water. Subsequently, seeds were transferred to a climate chamber to germinate for two days. 40 Replicate seedlings were transplanted to separate Rockwool blocks of 4 x 4 cm in a climate chamber (16h/8 h (day/night), 20/18°C (day/night) with light conditions of 125 μmolm^-2^s^-1^ and relative humidity of 70%). Replicates were divided over two different treatment groups: control (C; n = 20) and salt (S; n = 20). The plants were watered daily with 1/1000 Hyponex solution (C plants), or 50 mM NaCl 1/1000 Hyponex solution (S plants). Seeds were harvested and pooled per treatment at the end of the growth period. From these seed pools 40 seedlings (G.2) were grown per parental treatment which were subsequently divided over the two treatments (C, n = 20 and S, n = 20), resulting in four different experimental groups (Fig 1, G.2). This experimental design was repeated for three generations, in a full factorial design (Fig 1), resulting in eight groups with different parental histories coded as: CCC, CCS, CSC, CSS, SCC, SCS, SSC and SSS, with the first letter representing the treatment of the first generation (G.1) and so on.

### Offspring (G.4) evaluation in salt and control environment

Seeds (G.4) from all eight parental histories were placed on filter paper moistened with deionised water and stratified at 4°C in darkness for three days. After stratification three seeds were placed on top of a 1:4 mixture of pumice and potting soil, in 7 cm diameter pots. After 12 days, all but one randomly selected plant were removed. The experiment followed a blocked split-plot design, where the experimental treatment (S or C) was applied to groups of eight plants, in trays. Parental families were equally distributed among the trays. The trays were divided over six spatial blocks within a climate chamber, with five replicate trays of each treatment per spatial block. Parental histories were randomized within trays and trays were randomized within spatial blocks. The plants were grown at 16/8 h (day/night), 20/18°C (day/night) with light conditions of 120μM/cm^2^ and relative humidity of 70%. The pots were watered to saturation by flooding the trays three times a week for one hour, after which excess water was removed from the trays. Salt treatment was started 15 days after sowing following the same watering procedure, but with water containing 150mM NaCl. The treatment was stopped 39 days after germination after which the normal watering regime was resumed.

### Offspring (G.4) evaluation under field conditions

Seeds (G.4) from all eight parental histories were placed on filter paper moistened with deionised water. After three days of stratification at 4°C in darkness seeds were placed into a climate chamber to germinate for two days. The seedlings were then transplanted individually into 7 cm diameter net pots filled with a 1:1 mixture of potting soil and soil from the experimental field. Field soil was used to ensure that plants were exposed to natural biological soil interactions that are characteristic of the experimental field site. All plants were placed in an unheated glasshouse. In April 2012, 24 days after germination all plants were planted into an open field at the experimental gardens of the Netherlands Institute of Ecology, Wageningen, the Netherlands. The experiment followed a randomized block design with two replicates of each group per block and 18 replicate blocks, with individual plants placed at 10 cm intervals. The plants were watered with tap water one to three times a week during dry periods. All plants were harvested after three months.

### Measurements

In both the field and the climate chamber environments rosette diameter was measured 29 days after germination. Flowering time was recorded daily as the number of days from germination until opening of the first flower (all petals visible). The field plants were harvested approximately 11 weeks after transplantation when almost all plants had ceased flowering, started to senesce and mostly had fully matured siliques. The number of fruits per plant was counted as a measure of reproduction. The climate chamber plants were harvested after 11 weeks, approximately 7 weeks after the onset of flowering, when siliques were fully matured and the plants were well into senescence. Above ground biomass was determined after three days of oven drying at 70°C. Due to plant size it was not feasible to count the number of fruits for plants grown in the climate chamber, therefore we chose to use above ground biomass which often correlates strongly with number of fruits [28–31]. Because of their small size we could not accurately determine (initial) seed weights of the experimental plants individually. After setting up the experiments we determined average seed weight by weighing multiple sets of approximately 20 seeds for each of the experimental groups and we calculated average seed weight accordingly. For most of the experimental groups we weighed 8 sets of ~20 seeds, but because not enough seeds were available for all groups we could weigh only three sets of ~20 seeds for the CCS and SSC groups, and the SCS and CSS groups had to be excluded from the seed weight analysis altogether because <20 seeds were available.

### Statistical analysis

To test for the generational extent of environment-induced responses in *A*. *thaliana* we fitted generalized linear mixed- effect models for each offspring environment (control, salt and field) that estimated the effect of parental history (eight groups) on phenotypes, in which we performed several a priori contrast tests that we considered as the most relevant components of the overall parental (P), grandparental (GP) and great-grandparental (GGP) effects. Specifically to test for the presence of environmentally induced transgenerational responses we performed three different contrasts where the CCC group was compared to CCS, CSC and SCC to test for P, GP and GGP effects, respectively. Furthermore, and in addition to the a priori contrast tests, we tested for the presence of overall P, GP and GGP treatment effects on G.4 traits across the entire experimental design using generalized linear mixed-effect models for which the eight parental history groups (Fig 1) were recoded as a 2x2x2 factorial design with P, GP and GGP treatments as fixed factors. Block was included as a random factor in all models. In the controlled-environment salt and control experiments, a tray effect nested within blocks was also included as a random factor. Differences between experimental groups were analysed using 95% confidence intervals and p-values [32, 33]. The 95% confidence intervals of the effect sizes were calculated by parametric bootstrapping, and effects were labelled significant if the 95% CI of the effects size did not include zero. To account for multiple testing all *p*-values from the same environment were evaluated against an FDR threshold of 0.1 [34]. Seed weight was analysed using linear models, one where we performed similar contrasts as mentioned above and a separate model for which we recoded the parental history groups as the 2x2x2 factorial design.

To test for the effects of multiple-generation exposure to salt stress, which we refer to as ‘dose effects’, we fitted two different sets of a priori contrasts. First, we tested if the expression of the parental salt treatment effect was affected by GP and/or GGP salt treatment; i.e., H_0_: CCC-CCS = CSC-CSS = SSC-SSS (Dose effect 1). This hypothesis was tested by breaking it down into three comparisons: 1: CCC-CCS = CSC-CSS; 2: CSC-CSS = SSC-SSS and 3: CCC-CCS = SSC-SSS. If any of these comparisons showed a significant difference, we rejected the null hypothesis that the expression of the parental salt effect is unaffected by earlier salt treatments in the GP or GGP generations. Second, we tested if offspring of salt-stressed parents differed depending on the number of consecutive ancestral generations that were exposed to salt treatment; i.e., H_0_: CCS = CSS = SSS (Dose effect 2). This hypothesis was tested by splitting into two different comparisons: 1: CCS = CSS and 2: CSS = SSS.

To assess if the salt treatment influenced the expression of environment-induced transgenerational effects, the effects of salt treatment and their interaction with parental histories were analysed for the climate chamber data using linear mixed-effects model with offspring treatment (2 levels) and parental history (Fig 1), recoded as a 2x2x2 factorial design with parental (P), grandparental (GP) and great-grandparental (GGP) treatments as fixed factors, and block and tray effect nested within blocks included as random factors. Of special interest is the treatment*parental history effect as this would indicate a possible adaptive parental history effect.

In order to meet the assumption of homogeneity of variances and residual normality, number of fruits was ln-transformed and some incidental outliers were removed. Specifically, for diameter nine outliers, for flowering two outliers and for number of fruits six outliers were removed. Excluding these outliers never affected the significance of test results but removing the outliers resulted in a better model fit. In all models Gaussian error distribution with identity link function were used.

Note that our analysis considers individual plants as independent units; however, dependencies arise inevitably due to the pedigree structure (Fig 1) of our experiment. Due to this structure different experimental groups derive from the same grandparental or great-grandparental individuals. The statistical results are valid under the assumption that seed pools derived from each G3 group in the pedigree are an unbiased set whose properties differ in a representative way only due to the different treatments in their ancestral generations.

All analyses were performed in R version 3.1.2, using the lme4 package [35, 36].

## Results

### Effects of a single generation of salt exposure

**Fig 2:**
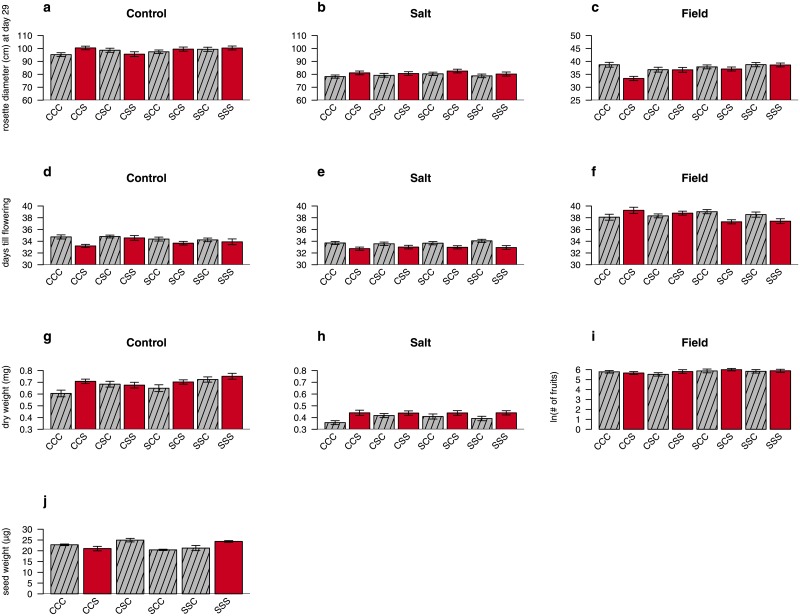
Expression of offspring phenotypes in three different environments after exposure of parental, grandparental and/or great-grandparental generations to salt stress. Panels a, b and c show rosette diameter (mm); panels d, e and f show flowering time (days); panels g, h, i show biomass (mg) or number of fruits (mean ± sem) for respectively climate chamber control, climate chamber salt and field environment. Panel j shows average seed weight, which only shows six experimental groups because of limited seed availability. Significant differences between groups are shown in Table 2. The striped grey bars indicate that the parents of the experimental plants were grown under control conditions. The red bars indicate that the parents of the experimental plants were grown under salt stress.

The effects of a single ancestral exposed generation were tested in two ways. First, by evaluating overall P, GP and GGP effects across the entire experimental design. Second, by comparing offspring of specific groups whose recent ancestors did not experience salt stress (CCC) to offspring of groups that were exposed to a single generation of salt stress either one generation ago (CCS; P effect), two generations ago (CSC; GP effect), or three generations ago (SCC; GGP effect). Strong parental effects were found in almost all traits and offspring environments for both approaches (Tables 1 and 2; for intercepts, unstandardized effect sizes and 95% confidence intervals see S1 and S2 Tables). Parental effects were typically among the strongest responses observed across the entire experimental design (P effect in Fig 2: CCC versus CCS bars). Grandparental and great-grandparental effects were weaker and less frequently found but still significant for seed weight, diameter and biomass (Tables 1 and 2).

**Table 1:** Results of linear model analysis of seed weight and generalized linear mixed-effects models for diameter, flowering time, dry weight and number of fruits, in which the experimental design is recoded as a 2x2x2 factorial with P, GP and GGP treatments as fixed effects. Shown are unstandardized effect sizes and *p*-values, significant values are indicated in bold. Each model was carried out separately for each offspring environment.

	Seed weight (mg)		Rosette diameter (mm)						Flowering time (days)						Dry weight (mg)				Ln(# Fruits)	
			Control		Salt		Field		Control		Salt		Field		Control		Salt		Field	
	Effect size	*p*-value	Effect size	*p*-value	Effect size	*p*-value	Effect size	*p*-value	Effect size	*p*-value	Effect size	*p*-value	Effect size	*p*-value	Effect size	*p*-value	Effect size	*p*-value	Effect size	*p*-value
Parent (P)	0.58	0.390	1.34	0.174	**2.00**	**0.013**	**-1.57**	**0.010**	**-0.70**	**0.002**	**-0.86**	**<0.001**	-0.32	0.270	**0.04**	**0.009**	**0.05**	**<0.001**	0.08	0.479
Grandparent (GP)	**2.73**	**<0.001**	0.42	0.673	-0.85	0.290	0.99	0.105	0.38	0.094	0.12	0.462	-0.17	0.558	**0.04**	**0.012**	0.01	0.396	-0.07	0.501
Great grandparent (GGP)	**-1.80**	**0.004**	1.67	0.091	0.72	0.371	**1.68**	**0.006**	-0.28	0.215	0.14	0.406	-0.53	0.069	**0.04**	**0.022**	0.007	0.569	0.20	0.072

**Table 2:** Results of the linear model (for seed weight) and generalized linear mixed-effects model analysis of *a priori* contrast tests. Each trait, except seed weight, was analysed separately per offspring (G.4) environment. For seed weight Dose effect 1 and Dose effect 2 could not be tested because some experimental groups had to be excluded due to not enough available seeds. Shown are unstandardized effect sizes and *p*-values, significant values are indicated in bold.

	Seed weight (mg)		Rosette diameter (mm)						Flowering time (days)						Dry weight (mg)				Ln(# Fruits)	
			Control		Salt		Field		Control		Salt		Field		Control		Salt		Field	
	Effect size	*p*-value	Effect size	*p*-value	Effect size	*p*-value	Effect size	*p*-value	Effect size	*p*-value	Effect size	*p*-value	Effect size	*p*-value	Effect size	*p*-value	Effect size	*p*-value	Effect size	*p*-value
P effect: CCC = CCS	-1.76	0.100	**5.17**	**0.007**	2.87	0.075	**-5.31**	**<0.001**	**-1.53**	**<0.001**	**-0.67**	**0.005**	1.20	0.039	**0.10**	**0.002**	**0.08**	**<0.001**	-0.11	0.589
GP effect: CCC = CSC	**2.16**	**0.008**	3.37	0.081	1.00	0.533	-1.91	0.111	0.07	0.883	-0.13	0.694	0.23	0.687	**0.08**	**0.018**	**0.06**	**0.014**	-0.26	0.234
GGP effect: CCC = SCC	**-2.37**	**0.004**	1.99	0.307	2.13	0.184	-0.83	0.486	-0.37	0.419	-0.03	0.922	0.93	0.099	0.04	0.179	0.06	0.033	0.08	0.712
Dose effect 1:																				
CCC-CCS = CSC-CSS	n/a	n/a	**-2.05**	**0.003**	-0.38	0.509	**1.31**	**0.002**	0.33	0.043	0.10	0.404	-0.19	0.354	**-0.02**	**0.019**	-0.02	0.070	0.10	0.209
CSC-CSS = SSC-SSS	n/a	n/a	1.03	0.132	0.03	0.951	-0.01	0.981	-0.03	0.876	-0.15	0.207	-0.39	0.056	0.01	0.450	0.007	0.433	-0.06	0.468
CCC-CCS = SSC-SSS	n/a	n/a	-1.02	0.137	-0.34	0.551	**1.30**	**0.002**	0.30	0.06	-0.05	0.666	**-0.58**	**0.005**	-0.02	0.110	-0.008	0.302	0.04	0.583
Dose effect 2:																				
CCS = CSS	n/a	n/a	1.59	0.154	0.44	0.634	**-2.86**	**<0.001**	**-0.69**	**0.009**	-0.14	0.469	**0.80**	**0.018**	-0.003	0.846	0.001	0.947	-0.11	0.363
CSS = SSS	n/a	n/a	-1.65	0.143	0.38	0.681	**-2.38**	**<0.001**	-0.01	0.966	-0.02	0.931	**1.07**	**0.001**	-0.04	0.041	-0.001	0.969	-0.09	0.440

### Effects of multiple generations of salt exposure

Two different methods were used to investigate whether exposure to salt stress for multiple generations is different from single-generation exposure. The first method tested if the expression of the parental salt treatment effect was affected by the salt treatment of grandparental and great-grandparental generations. This was observed for seed weight, rosette diameter in the field environments, for flowering in the field environment and for dry weight in the control and salt environment (Table 2, Dose effect 1). Second, we tested if offspring phenotypes were different between groups whose recent ancestors had been exposed to salt stress for one, two or three consecutive generations of stress exposure. Such dose effects (i.e. exposure of stress for multiple generations) were observed for diameter and flowering time in the field environment, and for flowering and dry weight in the control environment (Table 2, Dose effect 2; Fig 2). Both dose effects demonstrate that multiple generations of stress exposure had a different effect on offspring phenotype than a single generation of exposure. This indicates that plant traits were not only affected by parental environments but also by (great)grandparental environments. Often the parental effect was not amplified but instead reduced by multiple generations of stress exposure. Most notably, in the field environment the negative effect of parental salt exposure on offspring diameter and flowering growth and performance disappeared when also grandparents and great-grandparents had been exposed to salt stress (see Fig 2c and 2f; CCS vs CSS vs SSS bars).

### Effect of offspring environment

**Fig 3:**
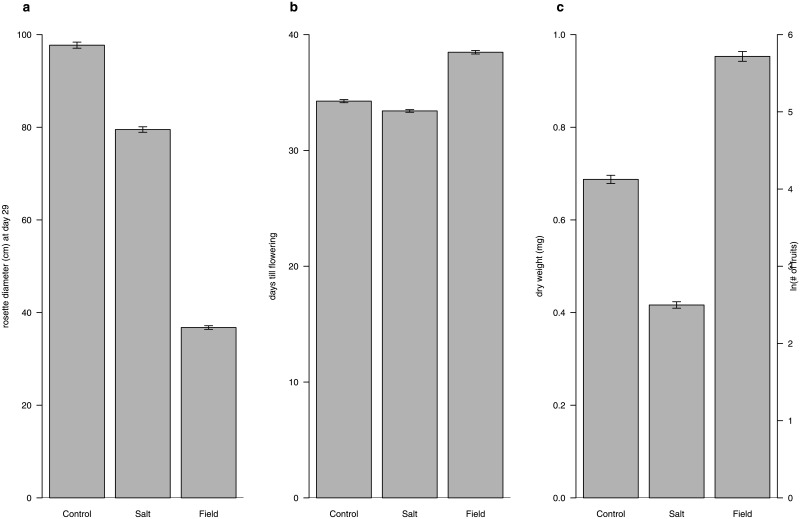
Mean and standard error of phenotypic traits measured in three environments. The bars represent the overall mean of all eight parental history groups separated per environment.

Offspring phenotype was strongly affected by the environment. Plants grown in the field had a 62% smaller rosette diameter compared to control plants grown in the climate chamber and were 54% smaller compared to salt treated plants in the climate chamber. Field-grown plants flowered 4 days later than climate chamber grown control plants and 5 days later than climate chamber grown salt treated plants. In the salt treatment plants had on average 40% less biomass compared to plants that were grown under control conditions (Fig 3).

In the climate chamber experiment, offspring from salt treated plants were larger and flowered earlier than offspring from control treated plants (Fig 3, control vs salt environments). These results were consistent in both the control and salt environment, although plants in the salt environment performed less well than plants in the control environment. No interaction effect between offspring treatment and parental treatment was found in the climate chamber experiment (Table 3; S3 Table shows the intercepts, unstandardized effect sizes, 95% confidence intervals). Indicating that parental or (great)grandparental exposure to salt did not result in a specific growth advantage under offspring salt stress. A significant parental effect of salt stress was also detected in the field experiment, but the direction of the effect was opposite compared to the climate chamber experiment. In the field environment offspring from control plants performed better, with larger rosette diameter and also earlier flowering than offspring from salt treated plants (Fig 2c and 2f, CCS). To explicitly test for the effect of offspring environment on the expression of transgenerational effects, data for rosette diameter and flowering time were z-transformed (within each offspring environment; see S4 Table). Significant parental ×offspring environment and great grandparental ×offspring environment interaction effect were observed for rosette diameter, as well as a marginally significant parental ×offspring environment and great grandparental ×environment interaction effect for flowering time interaction was found. These results confirm that offspring environment influences transgenerational effects. However, there was no evidence for adaptive transgenerational effects, which means that exposure to the ancestral treatment does not necessarily lead to a better offspring performance.

**Table 3:** Results of generalized linear mixed-effect model analysis of the climate chamber experiment, with (G.4) treatment (Control or Salt), parental history and the interaction between historic and test environment. Shown are unstandardized effect sizes and *p*-values, significant values are indicated in bold.

	Rosette diameter (mm)		Flowering time (days)		Dry weight (mg)	
	Effect size	*p*-value	Effect size	*p*-value	Effect size	*p*-value
Treatment	**-16.98**	**< 0.001**	**-0.93**	**0.005**	**-0.28**	**<0.001**
Parent (P)	1.30	0.158	**0.70**	**<0.001**	**0.04**	**0.006**
Grandparent (GP)	0.96	0.302	0.38	0.062	**0.04**	**0.008**
Great grandparent (GGP)	1.14	0.218	-0.28	0.167	**0.04**	**0.003**
Treatment * P	0.71	0.587	-0.10	0.735	0.003	0.848
Treatment * GP	-1.54	0.242	-0.20	0.486	-0.03	0.140
Treatment *GGP	-0.69	0.601	0.49	0.094	-0.03	0.097

## Discussion

Plant phenotype can be influenced by environments experienced by previous generations. Here we showed that these environment-induced parental and transgenerational effects are highly context dependent. Because of this context-dependency, the generality and importance of these effects has remained controversial. Our study has a unique design that enabled us to compare differences in responses, not only between offspring environments but also between single- and multiple-generations of exposure. We show that the expression of parental and transgenerational effects does not only depend on offspring environment, but is also strongly dependent on the number of ancestral generations that were exposed to environmental stress.

### Context-dependent expression of salt stress in *A*. *thaliana*

Boyko and colleagues (2010) showed that offspring from salt exposed transgenic *A*. *thaliana* C24 plants had higher recombination rates and also a higher tolerance to salt in the following generation [16]. An additional study on these transgenic lines showed differences in DNA methylation, histone modification and gene expression in offspring of the exposed plants [25]. In contrast, Suter & Widmer (2013) did not elicit parental or transgenerational effects after two consecutive generations of exposure of *A*. *thaliana* Col-0 plants to mild salt stress during vegetative growth [18], although improved growth under salt conditions was observed when plants were exposed for three consecutive generations and crossed with the Sha-0 genotype [17]. In our study, a relative high concentration of salt was administered to the plants throughout their entire lifetime, as well as to the G.4 offspring. Our results showed a strong parental effect of salt exposure in virtually all traits and in all three environments. Most studies focussing on transgenerational effects only tested single or multiple generations of exposure in a single environment [16–18, 25]. Our data revealed that offspring response after multi-generations of exposure to salt stress is different to the response after a single-generation of exposure. In addition, the expression of parental and transgenerational effects strongly depended on the evaluation environment which may explain the discrepancy between results of different studies that were performed with different conditions of stress inducement and evaluation environment.

### Parental effects

Our results provide strong evidence for the presence of parental effects. In our study parental salt treatment exposure extended during the entire life of the plants, including flowering and seed development. Thus, G.4 plants evaluated in the described experiments were already exposed to the parental treatment (salt or control) as developing embryos on the mother plant. Parental effects of salt stress as identified in this study therefore can include effects that were transmitted from the exposed parental plant as well as effects of direct induction of the G.4 as developing embryo. However, no direct parental effects on seed weight were found, which may be expected if the parental effects resulted from direct induction during seed development.

Interestingly the expression of parental effects interacts strongly with offspring environment (See S4 Table). For example, opposite directions of parental effects were observed between field and climate chamber environments. A speculative explanation for this difference may be related to possible parental effects on osmotic stress response [37]. A reduced response to osmotic stress can be a way to increase tolerance to soil salinity and results in greater leaf growth and stomata conductance, but only in the presence of sufficient pore water in the soil. If the system is water-limited this response could lead to depletion of the pore water before the plant is fully matured [37]. An important difference between the field and the growth chamber was that plants in the growth chamber had sufficient access to water, while field plants experienced episodes of drought stress and required additional watering during very dry periods. Most field plants showed purple leaves, presumably caused by anthocyanin, which is an indication of stress, including drought stress [38]. If a parental effect of salt stress acts via reduced offspring response to osmotic stress then this may reduce offspring performance in dry environments, while enhancing performance in wet environments. The observed differences between the field and the growth chamber show the importance of testing in more natural conditions, where different stressors interact and can significantly influence plant responses.

### Transgenerational effects

Plant phenotype was also partly determined by the environments that were experienced by grandparents or great-grandparents, even after a single generation of salt exposure. Contrary to expectations, the multiple generations of salt exposure did not amplify but instead tended to reduce the parental effect. This was most striking when offspring were evaluated in the field environment where both diameter and flowering time showed a reduced expression of the negative parental effect: parental salt exposure resulted in smaller and later-flowering plants, but these negative growth effects disappeared when not only parents but also (great)grandparents had been exposed to salt stress. This pattern suggests gradual acclimatization of the lineage to reduce the negative parental effects of salt stress. Some studies have previously reported that the heritable effects of multiple-generation exposure can differ substantially from single-generation parental exposure [23]. This may indicate that the gradual acclimatization is epigenetically mediated. Alternatively, gradual acclimatization may be the result of unintentional selection during the experimental pedigree. Our experimental design used bulked seed batches in the G.1-G.3 of the environmental treatments, and not single-seed descent, which inevitably biases seed batches to plants that performed well under the specific growing conditions. However, due to the highly inbred nature of Col-0 we think it is highly unlikely that the observed parental and transgenerational effects are due to genetic variation between plants. It is therefore more likely that the gradual acclimatization is epigenetically mediated.

### Adaptive value of parental and transgenerational effects

Parental and transgenerational effects in response to a given environmental stress are generally considered to be adaptive when offspring performance is enhanced under these conditions, but not under non-stressed conditions [16, 17]. Because we observed opposite responses to parental salt treatment in the climate chamber environments and the field environment it is difficult to conclude that the inherited response of salt stress is adaptive in our study. Parental effects may lead adaptive responses, irrespective of offspring environment, like enhanced growth and earlier flowering. Increased biomass may lead to a competitive advantage and increases the probability of successful establishment. This was suggested in *Medicago truncatula* plants, where parental plants were grown under salt stress leading to increased offspring size and enhancement of successful establishment of offspring plants [39]. In some situations it is possible that early flowering could be used as a mechanism to avoid environmental conditions that would otherwise increase mortality before the start of reproduction [37, 40]. Correspondingly in the salt treatment all plants flowered earlier when compared to control plants. However, it seems that this is not a general response to saline conditions in *A*. *thaliana* because other studies found later flowering after salt exposure [18, 41].

Additionally salt stress can influence root biomass, although root growth is usually less affected by salt stress than shoot growth [37]. In this study, plants were harvested at the end of their reproductive period when the majority of siliques had matured. Very likely, resources from both shoot and roots had been extracted and translocated to the seeds, and we therefore did not attempt to measure root biomass. We were thus unable to study the effects of transgenerational or direct salt exposure on root biomass and resource allocation. A study by Boyko and colleagues (2010) however showed longer root length in seedlings whose parents were exposed to saline conditions [26] indicating that transgenerational salt exposure may affect root biomass and resource allocation in *A*. *thaliana*.

### Underlying mechanisms

Our study does not address the underlying mechanisms that are responsible for the observed effects of ancestral environments. One possible mechanism of parental effects is the influence of seed size or seed quality [3, 11]. In our experiment, initial seed weight was affected by transgenerational environments, but not by parental environments, in six different experimental pre-treatment groups. Similar results were found in several studies (e.g. [18, 20]). Additionally initial seed weight did not show clear correlations with other traits such as rosette diameter, flowering time, dry weight or fruit number (See S1 Fig and S5 Table). Therefore it seems unlikely that seed size or seed quality are main determinants of the observed parental and transgenerational effects in these traits.

Epigenetic inheritance, mediated for instance by DNA methylation or small RNAs [11, 12, 42–45], may be a prime mechanism for the observed transgenerational effects, given that presumably no significant genetic variation was present in our *A*. *thaliana* Col-0 line. Epigenetic changes in response to salt treatment have been found in rice and *A*. *thaliana*, the latter also showing epigenetic modifications in offspring of exposed plants [25, 46]. Meiotic stability of part of such environment-induced epigenetic modifications could conceivably account for the observed transgenerational effects, although unequivocal evidence for such mechanism has been elusive [47].

## Conclusion

In our study we tried to gain a better insight in the context dependency of transgenerational effects by comparing single to multiple generations of ancestral exposure in different environments. Our results show that both the offspring environments and the number of ancestral generations that were exposed play a significant role in the phenotypic expression of these effects. The observed difference in the responses to different environments and different number of exposed generations provide new insights into the context dependency of transgenerational effects. The results contribute to a better understanding of the importance of transgenerational effects. Although transgenerational effects were weaker and less commonly observed than parental effects, evidence for phenotypes being affected by grandparental or even great-grandparental environments was observed in nearly all traits and environments. However, there are several general issues that remain unresolved. For instance, genotypic effects can significantly influence the expression of transgenerational effects [17, 18]. It remains however unclear to what extent our results are representative for other *A*. *thaliana* genotypes since only one genotype was tested in this study. It also remains to be demonstrated if, or to what extent, transgenerational effects are adaptive. It is possible that many transgenerational effects are merely caused by direct stress responses, for instance when epigenetic marks are not completely reset between generations, rather than being a possible adaptive response [47].

In conclusion, we showed that exposure to salt treatment can have persistent consequences for offspring phenotypes not only one generation after exposure, but also two or even three generations later. These transgenerational effects were often expressed as ‘dose’ effects where multiple-generation exposure has different impact on offspring traits than single-generation exposure. Our results suggest that transgenerational effects commonly occur and may have a considerable impact on plant phenotype, performance and the way plants respond to their environment.

## Supporting Information

S1 Fig Correlations between average seed weight and rosette diameter, flowering time, dry weight and number of siliques. (DOCX)

S1 Table Results of generalized linear mixed-effects model analysis of rosette diameter, flowering time, dry weight and number of fruits. (DOCX)

S2 Table Results of generalized linear mixed-effects model analysis of *a priori* contrast tests, each trait analysed separately per offspring (G.4) environment. (DOCX)

S3 Table Results of the generalized linear mixed-effect model analysis per trait for the climate chamber experiment, with (G.4) treatment (Control or Salt), parental history and the interaction between historic and test environment. (DOCX)

S4 Table Results of the generalized linear mixed-effect model analysis per trait for the climate chamber experiment, with (G.4) treatment (Control or Salt), parental history and the interaction between historic and test environment. (DOCX)

S5 Table Correlations between average seed weight and rosette diameter, flowering time, dry weight and number of siliques per offspring environment. (DOCX)
