# Supplementary material for: Effects of Multi-Generational Stress Exposure and Offspring Environment on the Expression and Persistence of Transgenerational Effects in Arabidopsis thaliana
Source: PLoS One. 2016 Mar 16;11(3):e0151566. doi: 10.1371/journal.pone.0151566 (PMC4794210; doi:10.1371/journal.pone.0151566)
Supplement: S1 Fig — (DOCX) [file pone.0151566.s001.docx]

Fig. S1. Correlations between average seed weight and rosette diameter, flowering time, dry weight and number of siliques. Correlations were done separately per environment. Fig. a, d and g show the control environment. The salt environment is shown in Fig. b, e and h. And the field environment is shown in Fig. c, f and i. Table S5 shows the r and *p*-values belonging to the correlation plots.
