# Supplementary material for: Effects of Multi-Generational Stress Exposure and Offspring Environment on the Expression and Persistence of Transgenerational Effects in Arabidopsis thaliana
Source: PLoS One. 2016 Mar 16;11(3):e0151566. doi: 10.1371/journal.pone.0151566 (PMC4794210; doi:10.1371/journal.pone.0151566)
Supplement: S1 Table — (DOCX) [file pone.0151566.s002.docx]

Table S1. Results of generalized linear mixed-effects model analysis of rosette diameter, flowering time, dry weight and number of fruits. Shown are the intercept, effect sizes, 95% confidence intervals; significant values are indicated in bold. Each model was carried out separately for trait and offspring environment.

|  | Rosette diameter (mm) | | | | | | | | | | |
| --- | --- | --- | --- | --- | --- | --- | --- | --- | --- | --- | --- |
|  | Control | | |  | Salt | | |  | Field | | |
|  | Effect size | 2.5% | 97.5% |  | Effect size | 2.5% | 97.5% |  | Effect size | 2.5% | 97.5% |
| Intercept | 96.57 | 93.60 | 99.67 |  | 79.21 | 75.64 | 82.57 |  | 36.70 | 35.38 | 38.06 |
| Parent (P) | 1.34 | -0.54 | 2.91 |  | **2.00** | **0.47** | **3.54** |  | **-1.57** | **-2.87** | **-0.26** |
| Grandparent (GP) | 0.42 | -1.73 | 2.44 |  | -0.85 | -2.52 | 0.82 |  | 0.99 | -0.30 | 2.19 |
| Great grandparent (GGP) | 1.67 | -0.30 | 3.56 |  | 0.72 | -0.79 | 2.24 |  | **1.68** | **0.41** | **2.95** |
|  | Flowering (days) | | | | | | | | | | |
|  | Control | | |  | Salt | | |  | Field | | |
|  | Effect size | 2.5% | 97.5% |  | Effect size | 2.5% | 97.5% |  | Effect size | 2.5% | 97.5% |
| Intercept | 34.48 | 34.00 | 34.98 |  | 33.62 | 32.95 | 34.28 |  | 38.85 | 38.18 | 39.51 |
| Parent (P) | **-0.7** | **-1.22** | **-0.29** |  | **-0.86** | **-1.12** | **-0.49** |  | -0.32 | -0.88 | 0.26 |
| Grandparent (GP) | 0.38 | -0.11 | 0.81 |  | 0.12 | -0.24 | 0.46 |  | -0.17 | -0.77 | 0.39 |
| Great grandparent (GGP) | -0.28 | -0.77 | 0.17 |  | 0.14 | -0.21 | 0.48 |  | -0.53 | -1.08 | 0.02 |
|  | Dry weight (mg) | | | | | | |  | Ln(#Fruits) | | |
|  | Control | | |  | Salt | | |  | Field | | |
|  | Effect size | 2.5% | 97.5% |  | Effect size | 2.5% | 97.5% |  | Effect size | 2.5% | 97.5% |
| Intercept | 0.63 | 0.59 | 0.66 |  | 0.38 | 0.35 | 0.42 |  | 5.69 | 5.46 | 5.91 |
| Parent (P) | **0.04** | **0.009** | **0.079** |  | **0.05** | **0.02** | **0.07** |  | 0.08 | -0.14 | 0.29 |
| Grandparent (GP) | **0.04** | **0.01** | **0.07** |  | 0.01 | -0.01 | 0.03 |  | -0.07 | -0.28 | 0.13 |
| Great grandparent (GGP) | **0.04** | **0.004** | **0.069** |  | 0.007 | -0.02 | 0.03 |  | 0.20 | -0.02 | 0.42 |
