# Supplementary material for: Effects of Multi-Generational Stress Exposure and Offspring Environment on the Expression and Persistence of Transgenerational Effects in Arabidopsis thaliana
Source: PLoS One. 2016 Mar 16;11(3):e0151566. doi: 10.1371/journal.pone.0151566 (PMC4794210; doi:10.1371/journal.pone.0151566)
Supplement: S2 Table — (DOCX) [file pone.0151566.s003.docx]

**Table S2. Results of generalized linear mixed-effects model analysis of *a priori* contrast tests, each trait analysed separately per offspring (G.4) environment.** Dose effects 1 and 2 test whether the effect of parental salt exposure differs depending on GP and GGP environments (see explanation in the Methods section). Shown are the intercepts, effect sizes and 95% confidence intervals; significant values are indicated in bold.

|  | Rosette diameter (mm) | | | | | | | | | | |
| --- | --- | --- | --- | --- | --- | --- | --- | --- | --- | --- | --- |
|  | Control | | |  | Salt | | |  | Field | | |
|  | Effect size | 2.5% | 97.5% |  | Effect size | 2.5% | 97.5% |  | Effect size | 2.5% | 97.5% |
| Intercept | 95.23 | 91.44 | 98.74 |  | 78.23 | 74.50 | 82.05 |  | 38.70 | 36.87 | 40.49 |
| P effect: CCC=CCS | **5.17** | **1.31** | **8.93** |  | 2.87 | -0.19 | 6.29 |  | **-5.31** | **-7.84** | **-2.67** |
| GP effect: CCC=CSC | 3.37 | -0.69 | 7.72 |  | 1.00 | -1.75 | 4.39 |  | -1.91 | -4.58 | 0.46 |
| GGP effect: CCC=SCC | 1.99 | -2.06 | 6.07 |  | 2.13 | -1.07 | 5.49 |  | -0.83 | -3.27 | 1.34 |
| Dose effect 1: |  |  |  |  |  |  |  |  |  |  |  |
| Intercept | 98.28 | 95.84 | 100.92 |  | 80.15 | 77.37 | 83.34 |  | 37.23 | 36.57 | 37.87 |
| CCC-CCS=CSC-CSS | **-2.05** | **-3.34** | **-0.67** |  | -0.38 | -1.58 | 0.73 |  | **1.31** | **0.50** | **2.24** |
| CSC-CSS=SSC-SSS | 1.03 | -0.344 | 2.38 |  | 0.03 | -1.26 | 1.23 |  | -0.01 | -0.84 | 0.90 |
| CCC-CCS=SSC-SSS | -1.02 | -2.35 | 0.34 |  | -0.34 | -1.35 | 0.80 |  | **1.30** | **0.51** | **2.19** |
| Dose effect 2: |  |  |  |  |  |  |  |  |  |  |  |
| CCS=CSS | 1.59 | -0.58 | 3.80 |  | 0.44 | -1.39 | 2.16 |  | **-2.86** | **-4.16** | **-1.44** |
| CSS=SSS | -1.65 | -3.89 | 0.43 |  | 0.38 | -1.56 | 1.96 |  | **-2.38** | **-3.65** | **-1.02** |
|  | Flowering time (days) | | | | | | | | | | |
|  | Control | | |  | Salt | | |  | Field | | |
|  | Effect size | 2.5% | 97.5% |  | Effect size | 2.5% | 97.5% |  | Effect size | 2.5% | 97.5% |
| Intercept | 34.73 | 34.08 | 35.41 |  | 33.7 | 32.93 | 34.46 |  | 38.10 | 37.22 | 38.94 |
| P effect: CCC=CCS | **-1.53** | **-2.41** | **-0.66** |  | **-0.67** | **-1.65** | **-0.26** |  | **1.20** | **0.06** | **2.40** |
| GP effect: CCC=CSC | 0.07 | -0.82 | 0.94 |  | -0.13 | -8.21 | 0.54 |  | 0.23 | -0.86 | 1.40 |
| GGP effect: CCC=SCC | -0.37 | -1.32 | 0.60 |  | -0.03 | -6.45 | 0.56 |  | 0.93 | -0.17 | 1.97 |
| Dose effect 1: |  |  |  |  |  |  |  |  |  |  |  |
| Intercept | 34.18 | 33.84 | 34.50 |  | 33.32 | 32.66 | 33.97 |  | 38.35 | 38.00 | 38.73 |
| CCC-CCS=CSC-CSS | 0.33 | -0.01 | 0.64 |  | 0.10 | -1.18 | 0.33 |  | -0.19 | -0.60 | 0.20 |
| CSC-CSS=SSC-SSS | -0.03 | -0.37 | 0.30 |  | -0.15 | -3.67 | 0.10 |  | -0.39 | -0.74 | 9.96 |
| CCC-CCS=SSC-SSS | 0.3 | -0.01 | 0.61 |  | -0.05 | -3.05 | 0.21 |  | **-0.58** | **-1.01** | **-0.19** |
| Dose effect 2: |  |  |  |  |  |  |  |  |  |  |  |
| CCS=CSS | **-0.69** | **-1.18** | **-0.22** |  | -0.14 | -4.93 | 0.24 |  | **0.80** | **0.07** | **1.44** |
| CSS=SSS | -0.01 | -0.56 | 0.53 |  | -0.02 | -4.00 | 0.36 |  | **1.07** | **0.42** | **1.69** |
|  | Dry weight (mg) | | | | | | |  | Ln(#Fruits) | | |
|  | Control | | |  | Salt | | |  | Field | | |
|  | Effect size | 2.5% | 97.5% |  | Effect size | 2.5% | 97.5% |  | Effect size | 2.5% | 97.5% |
| Intercept | 0.61 | 0.55 | 0.66 |  | 0.36 | 0.31 | 0.40 |  | 5.78 | 5.44 | 6.08 |
| P effect: CCC=CCS | **0.10** | **0.04** | **0.17** |  | **0.08** | **0.04** | **0.13** |  | -0.11 | -0.53 | 0.28 |
| GP effect: CCC=CSC | **0.08** | **0.01** | **0.15** |  | **0.06** | **0.013** | **0.10** |  | -0.26 | -0.71 | 0.22 |
| GGP effect: CCC=SCC | 0.04 | -0.03 | 0.11 |  | **0.06** | **0.004** | **0.10** |  | 0.08 | -0.35 | 0.53 |
| Dose effect 1: |  |  |  |  |  |  |  |  |  |  |  |
| Intercept | 0.69 | 0.67 | 0.71 |  | 0.42 | 0.39 | 0.45 |  | 5.79 | 5.64 | 5.93 |
| CCC-CCS=CSC-CSS | **-0.02** | **-0.05** | **-0.003** |  | **-0.02** | **-0.03** | **-1e-05** |  | 0.10 | -0.05 | 0.23 |
| CSC-CSS=SSC-SSS | 0.01 | -0.01 | 0.03 |  | 0.007 | -0.008 | 0.02 |  | -0.06 | -0.22 | 0.12 |
| CCC-CCS=SSC-SSS | -0.02 | -0.04 | 0.01 |  | -0.008 | -0.025 | 0.007 |  | 0.04 | -0.12 | 0.19 |
| Dose effect 2: |  |  |  |  |  |  |  |  |  |  |  |
| CCS=CSS | -0.003 | -0.04 | 0.03 |  | 0.001 | -0.027 | 0.03 |  | -0.11 | -0.39 | 0.15 |
| CSS=SSS | **-0.04** | **-0.08** | **-0.003** |  | -0.001 | -0.031 | 0.03 |  | -0.09 | -0.34 | 0.15 |
