# Supplementary material for: Effects of Multi-Generational Stress Exposure and Offspring Environment on the Expression and Persistence of Transgenerational Effects in Arabidopsis thaliana
Source: PLoS One. 2016 Mar 16;11(3):e0151566. doi: 10.1371/journal.pone.0151566 (PMC4794210; doi:10.1371/journal.pone.0151566)
Supplement: S3 Table — (DOCX) [file pone.0151566.s004.docx]

**Table S3. Results of the generalized linear mixed-effect model analysis per trait for the climate chamber experiment, with (G.4) treatment (Control or Salt), parental history and the interaction between historic and test environment.** Shown are the intercepts, effect sizes, 95% confidence intervals; significant values are indicated in bold.

|  | Rosette diameter (mm) | | |  | Flowering time (days) | | |  | Dry weight (mg) | | |
| --- | --- | --- | --- | --- | --- | --- | --- | --- | --- | --- | --- |
|  | Effect size | 2.5% | 97.5% |  | Effect size | 2.5% | 97.5% |  | Effect size | 2.5% | 97.5% |
| Treatment | **-16.98** | **-19.95** | **-13.86** |  | **-0.93** | **-1.46** | **-0.29** |  | **-0.28** | **-0.28** | **-0.20** |
| Parent (P) | 1.30 | -4.65 | 3.16 |  | **0.70** | **-1.10** | **-0.29** |  | **0.04** | **0.01** | **0.07** |
| Grandparent (GP) | 0.96 | -7.29 | 2.64 |  | 0.38 | -1.49 | 0.78 |  | **0.04** | **0.01** | **0.07** |
| Great grandparent (GGP) | 1.14 | -6.61 | 2.82 |  | -0.28 | -6.87 | 0.15 |  | **0.04** | **0.01** | **0.07** |
| Treatment * P | 0.71 | -2.01 | 3.03 |  | -0.10 | -6.56 | 0.47 |  | 0.003 | -0.004 | 0.06 |
| Treatment * GP | -1.54 | -4.13 | 0.98 |  | -0.20 | -7.53 | 0.36 |  | -0.03 | -0.07 | 0.01 |
| Treatment *GGP | -0.69 | -3.47 | 1.68 |  | 0.49 | -8.07 | 1.09 |  | -0.03 | -0.07 | 0.004 |
