# Supplementary material for: Effects of Multi-Generational Stress Exposure and Offspring Environment on the Expression and Persistence of Transgenerational Effects in Arabidopsis thaliana
Source: PLoS One. 2016 Mar 16;11(3):e0151566. doi: 10.1371/journal.pone.0151566 (PMC4794210; doi:10.1371/journal.pone.0151566)
Supplement: S4 Table — (DOCX) [file pone.0151566.s005.docx]

Table S4. Results of the linear mixed-effect model analysis for rosette diameter and flowering time for all three (Control, Salt and Field) offspring environments. Significant values are indicated in bold. Within each offspring environment, the data was normalised to the standardised z-scores in order to facilitate comparison of traits that were measured in different offspring environments.

|  | Rosette diameter (mm) | | | |  | Flowering time (days) | | | |
| --- | --- | --- | --- | --- | --- | --- | --- | --- | --- |
|  | numDF | denDF | F-value | *p*-value |  | numDF | denDF | F-value | *p*-value |
| Offspring environment | **2** | **683** | **291** | **<0.001** |  | **2** | **705** | **1497** | **<0.001** |
| Parent (P) | 1 | 683 | 1.10 | 0.295 |  | **1** | **705** | **22.8** | **<0.001** |
| Grandparent (GP) | 1 | 683 | 1.71 | 0.192 |  | 1 | 705 | 1.39 | 0.239 |
| Great grandparent (GGP) | **1** | **683** | **7.61** | **0.006** |  | 1 | 705 | 1.22 | 0.269 |
| Offspring environment * P | 2 | 683 | 8.39 | **<0.001** |  | 2 | 705 | 2.72 | 0.067 |
| Offspring environment * GP | 2 | 683 | 1.66 | 0.190 |  | 2 | 705 | 1.34 | 0.263 |
| Offspring environment *GGP | **2** | **683** | **3.94** | **0.020** |  | 2 | 705 | 2.21 | 0.110 |
