# Supplementary material for: Effects of Multi-Generational Stress Exposure and Offspring Environment on the Expression and Persistence of Transgenerational Effects in Arabidopsis thaliana
Source: PLoS One. 2016 Mar 16;11(3):e0151566. doi: 10.1371/journal.pone.0151566 (PMC4794210; doi:10.1371/journal.pone.0151566)
Supplement: S5 Table — (DOCX) [file pone.0151566.s006.docx]

Table S5. Correlations between average seed weight and rosette diameter, flowering time, dry weight and number of siliques per offspring environment.

|  | Seed weight | | |
| --- | --- | --- | --- |
|  | Offspring environment | r | *p*-value |
| Rosette diameter | Control | 0.060 | 0.91 |
|  | Salt | -0.290 | 0.58 |
|  | Field | 0.497 | 0.32 |
| Flowering time | Control | 0.389 | 0.45 |
|  | Salt | -0.159 | 0.76 |
|  | Field | -0.778 | 0.07 |
| Dry weight | Control | 0.178 | 0.74 |
|  | Salt | 0.123 | 0.82 |
| #Fruits | Field | -0.391 | 0.44 |
